# Successful treatment of AML using non-intensive chemotherapy in Jehovah's Witness patients

**DOI:** 10.1016/j.lrr.2024.100477

**Published:** 2024-08-08

**Authors:** David Page, Daniel Sawler, Joseph Brandwein

**Affiliations:** Division of Hematology, Department of Medicine, University of Alberta, Edmonton Alberta Canada

**Keywords:** Jehovah's Witness, AML, Leukemia, Chemotherapy, Treatment

## Abstract

Acute myeloid leukemia (AML) patients undergoing induction chemotherapy receive transfusion support to manage severe cytopenias and associated sequelae. Jehovah's Witness (JW) patients typically decline transfusion of most or all blood products. This can lead to exclusion of JW patients from otherwise life-saving treatments due to safety concerns. We present two cases demonstrating the successful induction of JW patients without the need for red cell or platelet transfusion support; the first, an older AML patient induced with azacitidine & venetoclax; the second, a patient with acute promyelocytic leukemia induced using arsenic trioxide and all-trans retinoic acid. Both patients required modifications to the induction regimens to accommodate their wishes. These cases support growing evidence that selected JW patients with AML can be successfully treated using appropriate accommodations.

## 1 Background

The Jehovah's Witness (JW) religious movement forbids the transfusion of blood products [1], although there are patient-to-patient differences. Some will accept plasma-derived products and not cells, while others will decline all blood products. This presents a major challenge when treating patients with acute myeloid leukemia (AML), who typically present with severe cytopenias.

The standard induction chemotherapy for AML consists of cytarabine plus an anthracycline [2]. This is typically associated with 4 weeks of marrow aplasia, requiring multiple red cell and platelet transfusions [2]. Even if non-intensive chemotherapy is given, patients typically require transfusions due to effects of the AML and concomitant chemotherapy. Despite rare reports of successful treatment without blood product support using non-intensive therapy [[3], [4]], JW patients have been reported to have inferior outcomes with chemotherapy [5], and many physicians are apprehensive administering myelosuppressive therapy to these patients. A new non-intensive AML induction regimen has recently become available, using a combination of the hypomethylating agent azacitidine plus the BCL2 inhibitor venetoclax (aza-ven) [6]. Although less intensive than standard induction chemotherapy, it is also myelosuppressive, and most patients receive transfusion support during the induction phase.

Acute promyelocytic leukemia (APL) is a rare AML variant characterized by the production of a novel PML-RARA protein and is associated with disseminated intravascular coagulation (DIC) and high bleeding risk. Treatment of lower risk patients with APL typically consists of the combination of all-trans retinoic acid (ATRA) and arsenic trioxide (ATO) [7]. Although remission and cure rates are high nearly all patients also receive blood products during the induction phase [8].

We report 2 cases of JW patients treated successfully - an AML patient treated with aza-ven and an APL patient treated with ATRA & ATO, each respecting patient wishes regarding transfusion support. Prior to publication consent from patients was obtained.

## 2 Case presentations

Case #1: A 76-year-old male JW patient, previously healthy, presented with a one-month history of exertional dyspnea. The initial complete blood count (CBC) showed a white blood count (WBC) 1.2 × 10^9^/L, hemoglobin (Hgb) 105 g/L, and platelet count 77 × 10^9^/L. The differential showed an absolute neutrophil count (ANC) of 0.1 × 10^9^/L and a rare circulating blast. Bone marrow biopsy revealed 49% blasts, CD13+, CD33+, CD117+ and TdT+, with weak CD5 expression; myeloperoxidase was negative. Cytogenetics showed a normal male karyotype, and mutational profiling by next generation sequencing identified ETV6, RUNX1 and STAG2 mutations. Chemistries, including liver enzymes, bilirubin, lactate dehydrogenase, creatinine, phosphate and albumin were normal, as were coagulation tests.

The patient declined transfusion of any blood products, even in the setting of life-threatening cytopenias. After discussion of the risks he consented to and received induction treatment with azacitidine 75 mg/m^2^ subcutaneously daily for 7 days, plus venetoclax 50 mg PO daily for 10 days. The venetoclax dose was reduced due to strong CYP3A4 interaction and duration reduced to minimize the myelosuppressive effects. He also received antimicrobial prophylaxis with posaconazole 300 mg PO daily, levofloxacin 500 mg PO daily and valacyclovir 500 mg PO daily. Hematinic support was provided with epoetin 40,000 units subcutaneously twice weekly, daily oral ferrous gluconate, vitamin B12 and folic acid. He was managed as an outpatient with once weekly bloodwork.

**Fig. 1:**
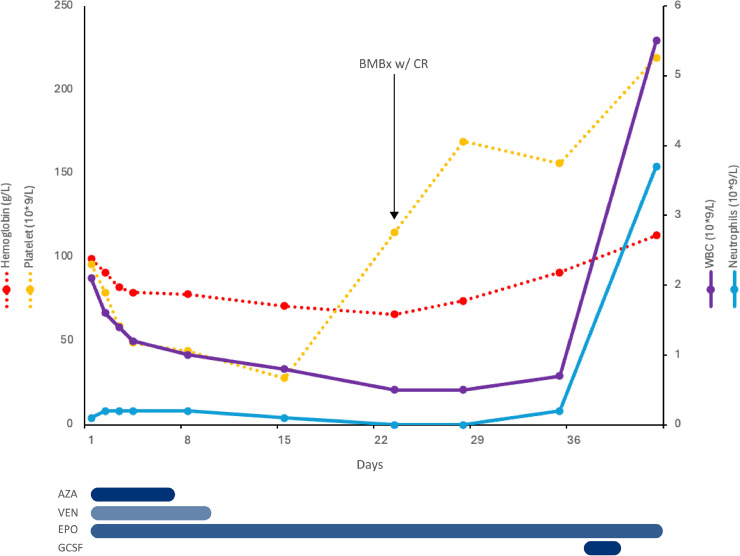
– Blood counts and supportive medications from start of induction for case #1. Aza = azacitidine; ven = venetoclax; EPO = epoetin; BMBx = bone marrow biopsy; CR = complete remission.

The hemoglobin nadir was 66 g/L on Day 22, while the platelet nadir was 28 × 10^9^/L on Day 15 (Fig. 1). The cytopenias subsequently improved without receiving blood products. Except for transient moderate generalized fatigue and exertional dyspnea while severely anemic he remained well and ambulatory, without bleeding issues. A bone marrow aspirate on Day 22 showed 1% blasts by morphology, consistent with morphologic remission; MRD testing was not performed. Peripheral blood counts fully recovered and antibiotics were stopped by Day 28 and hematinics were all stopped on Day 42. He subsequently began his 2nd treatment cycle, using venetoclax 400 mg PO daily x 14 days and azacitidine at the same doses as above. To date, he has completed 9 treatment cycles at the same azacitidine doses and venetoclax reduced to 10 days per cycle, administered every 5–6 weeks. He has required intermittent granulocyte colony-stimulating factor (G-CSF) for neutropenia but hemoglobin and platelet counts have remained stable, with no circulating blasts.

Case #2: A generally healthy 46-year-old female JW patient presented to the emergency department with a three-day history of fatigue and heavy menstrual bleeding. The initial CBC showed WBC 2.9 × 10^9^/L, Hgb 136 g/L, platelets 25 × 10^9^/L. The differential showed ANC 1.3 × 10^9^/L and absolute blast count of 0.4 × 10^9^/L. The fibrinogen was 0.5 g/L, INR 1.4, PTT 34 s, and d-Dimer 6.43 mg/L FEU. Molecular studies revealed PML-RARA and FLT3-ITD transcripts by PCR, and cytogenetics showed t(15;17) and trisomy 8. Bone marrow biopsy confirmed acute promyelocytic leukemia with 64% bone marrow blasts, CD13+, CD33+, CD34+, CD45+, CD117+, CD4+, CD19+ (dim), HLA-DR.-, CD11b-, CD2-, CD56-. A moderate-sized hematoma later developed at the biopsy site.

The patient agreed to the infusion of fractionated plasma-derived products but not cellular products or plasma, and accordingly was supported with fibrinogen concentrate to maintain fibrinogen levels ≥ 2.0 g/L throughout her course.

Hematinic support was provided with iron sucrose 300 mg intravenously daily for 3 days then weekly for 4 doses, folic acid, vitamin B12 and darbepoetin 300mcg subcutaneously 3 times weekly for 6 weeks. Where feasible bloodwork was drawn in pediatric tubes. Although initial labs suggested DIC, the patient was placed on tranexamic acid and high dose oral progesterone. With these measures the heavy menstrual bleeding resolved. No thrombotic complications occurred.

**Fig. 2:**
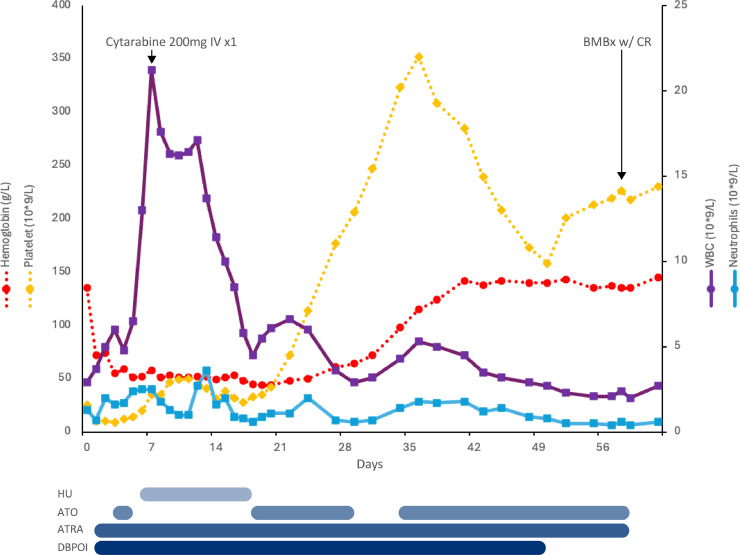
– Blood counts and supportive medications from start of induction for case #2. HU = hydroxyurea; ATO = arsenic trioxide; ATRA = all-trans retinoic acid; DBPOI = darbepoetin; BMBx = bone marrow biopsy; CR = complete remission.

ATRA 45 mg/m^2^ daily was initiated on Day 1, and ATO 0.15 mg/kg/day IV was initiated on Day 3. ATO was held at various points during induction (Fig. 2) for leukocytosis to minimize differentiation syndrome risk. Leukocytosis was managed with cytarabine 200 mg IV on day 7 and hydroxyurea up to 4 gs daily between Days 6–18. The Hgb nadir was 44 g/L on Day 19, and platelet nadir was 9 × 10^9^/L on Day 3, with a secondary platelet nadir of 28 × 10^9^/L on Day 17 (Fig. 2). The admission was complicated by a syncopal episode with hypotension and rigors, with urine culture positive for *E. coli*. Following prompt institution of antibiotics no further episodes occurred.

After receiving the equivalence of 30 days of full-dose ATO a bone marrow aspirate was performed on day 58, despite ongoing neutropenia, which revealed morphological remission and undetectable PML-RARA transcript levels. She then received 4 cycles of full-dose consolidation with ATRA and ATO, as described [7], with no further cytopenias or other issues. An end-of treatment bone marrow confirmed complete molecular remission with undetectable PML-RARA. The patient remains in molecular remission on peripheral-blood testing at last follow-up, 15 months post end-of-treatment.

## 3 Discussion

Our cases provide further evidence that JW patients with AML can be successfully treated with reduced intensity regimens, and should not be denied such treatment if they are otherwise medically fit, and made aware of the increased risks associated with treatment. These patients should be provided with maximal non-blood product supports, which is previously well-summarized [9]. As cell counts decline further during induction, owing to the combined effects of the underlying disease and the treatment-induced myelosuppression, interventions should be initiated quickly to minimize duration of life-threatening cytopenias.

Evidence for use of aza-ven in 2 JW patients has been published previously [4], although those were much younger patients. Our case #1 indicates that older patients may also benefit from this strategy. Limiting the duration of venetoclax exposure may reduce the severity of the post-induction cytopenias without compromising efficacy, although that has not been shown prospectively. Although not proven, supportive care with hematinic supplementation and erythropoietin stimulating agents may have aided in limiting the severity of the anemia.

Patients with APL are at high risk of early mortality relating to coagulopathy and/or sequelae of differentiation syndrome. Our case #2 demonstrates that, with the use of ATRA/ATO, these patients may be safely managed through induction. Intermittent cytoreduction may be necessary for leukocytosis to minimize differentiation syndrome risk, and contributes to worsening cytopenias, however as demonstrated this can be administered judiciously and safely. Invasive procedures should be delayed pending improvement in coagulopathy, if possible. Our patient was fortunately amenable to fibrinogen concentrate, which serves as a reminder JW patients are not homogeneous in their acceptance or refusal of blood-products, and careful review with each individual is warranted.

## Informed consent

Per the manuscript the patients provided informed consent. Per the Leukemia Research Report website we have not uploaded the patient consent forms at the present time.

## CRediT authorship contribution statement

**David Page:** Writing – review & editing, Writing – original draft, Visualization. **Daniel Sawler:** Writing – review & editing. **Joseph Brandwein:** Writing – review & editing, Writing – original draft, Conceptualization.

## Declaration of competing interest

The authors declare the following financial interests/personal relationships which may be considered as potential competing interests:

Joseph Brandwein reports a relationship with AbbVie Inc that includes: board membership and consulting or advisory. Joseph Brandwein reports a relationship with Amgen Inc that includes: board membership and consulting or advisory. Joseph Brandwein reports a relationship with Astellas Pharma Inc that includes: board membership and consulting or advisory. Joseph Brandwein reports a relationship with Bristol Myers Squibb Co that includes: board membership and consulting or advisory. Joseph Brandwein reports a relationship with Jazz Pharmaceuticals Inc that includes: board membership and consulting or advisory. Joseph Brandwein reports a relationship with Pfizer that includes: board membership and consulting or advisory. Joseph Brandwein reports a relationship with Taiho Pharma Canada Inc that includes: board membership and consulting or advisory. If there are other authors, they declare that they have no known competing financial interests or personal relationships that could have appeared to influence the work reported in this paper.
